# Prediction of drug–ABC-transporter interaction — Recent advances and future challenges☆

**DOI:** 10.1016/j.addr.2015.03.001

**Published:** 2015-03-11

**Authors:** Floriane Montanari, Gerhard F. Ecker

**Affiliations:** University of Vienna, Department of Pharmaceutical Chemistry, Althanstrasse 14, 1090 Wien, Austria

**Keywords:** ABC transporters, Computational models, Bioassays, Machine learning, Pharmacophore modeling, Transport inhibition

## Abstract

With the discovery of P-glycoprotein (P-gp), it became evident that ABC-transporters play a vital role in bioavailability and toxicity of drugs. They prevent intracellular accumulation of toxic compounds, which renders them a major defense mechanism against xenotoxic compounds. Their expression in cells of all major barriers (intestine, blood–brain barrier, blood–placenta barrier) as well as in metabolic organs (liver, kidney) also explains their influence on the ADMET properties of drugs and drug candidates. Thus, *in silico* models for the prediction of the probability of a compound to interact with P-gp or analogous transporters are of high value in the early phase of the drug discovery process. Within this review, we highlight recent developments in the area, with a special focus on the molecular basis of drug–transporter interaction. In addition, with the recent availability of X-ray structures of several ABC-transporters, also structure-based design methods have been applied and will be addressed.

## 1 Introduction

ATP-binding cassette transporters (ABC-transporters) form a large superfamily of membrane proteins. Members of the ABC-transporters can be found in all living organisms from prokaryotes to mammals. Generally speaking, these transporters participate in active transport, *i.e.* they hydrolyze ATP and use its energy to transport their substrates. In humans, 49 ABC-transporters are recognized to date and belong to 7 distinct subfamilies [1], ABCA to ABCG. The usual “transport unit” consists of two intracellular nucleotide binding domains and two transmembrane domains. The nucleotide binding domains (NBDs), usually well conserved across subfamilies, bind and hydrolyze ATP. The transmembrane domains create the translocation chamber across which the substrates diffuse. These regions are usually little conserved and are responsible for the substrate specificity of the different transporters. Members of the ABCBA subfamily transport cholesterol and lipids [2]. Members of the B, C and G subfamilies are multi-drug resistance-associated transporters or associated with diseases.

It was in 1976 when Juliano and Ling [3] linked the phenomenon of anticancer multiple drug resistance to a single glycoprotein expressed in the membranes of Chinese hamster ovary cells. As multiple drug resistance was characterized by a decreased accumulation of the anticancer agents in the tumor cells, they named the protein P-glycoprotein (P for permeability). Soon after, it became evident that P-glycoprotein (P-gp) functions as an ATP-driven, transmembrane efflux pump with an extremely broad substrate specificity (polyspecificity or promiscuity). Obviously, there were immediate attempts to develop compounds which would block the P-gp mediated efflux of anticancer drugs and thus resensitize multidrug resistant tumor cells. The first representative of this new class of so-called modulators of multidrug resistance (MDR-modulators) was the calcium channel blocker verapamil [4,5]. As for substrates, also in case of inhibitors P-gp is characterized by an extremely broad ligand profile. Thus, there are currently more than 5000 compounds retrieved when you search the Open PHACTS Discovery Platform [6] for compounds interacting with P-glycoprotein. Several compounds were subject to clinical studies, but none was approved so far. This raised the question of the druggability of P-gp, and the research focus shifted towards its potential role as antitarget [7].

### 1.1 ABC-transporters and ADMET

With the increasing knowledge on the tissue expression and function of P-glycoprotein, its important role in absorption of drugs and drug candidates became evident. This is now broadly accepted and has been also picked up by regulatory authorities. Based on a proposal from the International Transporter Consortium, the FDA now recommends a standardized set of experiments to assess the likelihood of a compound to interact with P-glycoprotein and the Breast Cancer Resistance Protein (BCRP/ABCG2) [8], another member of this super-family of ABC-transporters. According to the multiple roles of P-gp and analogs, both substrate and inhibitor properties of compounds need to be explored. The latter especially is important for drug–drug interactions. There are numerous cases reported where co-administration of a P-gp inhibitor with a P-gp substrate considerably increased the blood levels of the latter, leading to serious side effects. Classical examples are drug–drug interactions with digoxin (dronedarone, quinidine, ranolazine), loperamide (tipranavir, ritonavir), saquinavir (tipranavir, ritonavir) for P-gp and interactions with topotecan (GF120918) for BCRP.

Compounds inducing expression of P-gp will lead to analogous results. However, as clearly exemplified in the Biopharmaceutics Classification System (BCS) [9], the solubility of the compounds plays also an indispensable role for assessing the final risk for transporter-related low bioavailability. As P-gp is an ATP-driven transporter, its transport capacity has limits and it can be saturated. P-gp does not play any role in the bioavailability of highly soluble compounds, irrespective of whether they are substrates or not. P-gp becomes the limiting step only for substrates with low solubility. This of course increases the complexity and renders the task of predicting bioavailability of a compound by *in silico* models quite a challenge.

The blood–brain barrier (BBB) has been recognized as a tissue/barrier where P-gp and BCRP play a major role in controlling the transcellular flux of small molecules. The BBB is characterized by tight junctions, which force all solutes to take the transcellular route. Both P-gp and BCRP are highly expressed at the BBB and thus are the major functional constituents of this barrier. This has implications for the development of CNS-active drugs, as these need to cross the BBB, and thus should be devoid of P-gp and BCRP substrate properties. Especially for the therapy of brain tumours, this is of major relevance, as most anticancer agents are substrates of P-gp and BCRP [10,11]. Very recent examples are provided by the group of Schinkel, who demonstrate that brain accumulation of the PARP inhibitor rucaparib and the JAK1/2 inhibitor CYT387 in mice is restricted by Abcg2 and Abcb1a/1b [12,13]. In contrast, for compounds supposed not to interact with CNS-targets, favoring P-gp substrate properties would be a versatile approach for preventing them from entering the brain. A classical example in this respect is the class of antihistaminic agents: the first generation of compounds (*e.g.* diphenhydramine) showed remarkable CNS-related side effects, such as dizziness, whereas the 3rd generation of drugs, such as fexofenadine, is devoid of CNS side effects due to their P-gp substrate properties [14]. In addition, as already outlined previously, drug–drug interactions mediated by P-gp and BCRP are an important issue also at the BBB [15].

### 1.2 ABC-transporters and liver toxicity

**Fig. 1:**
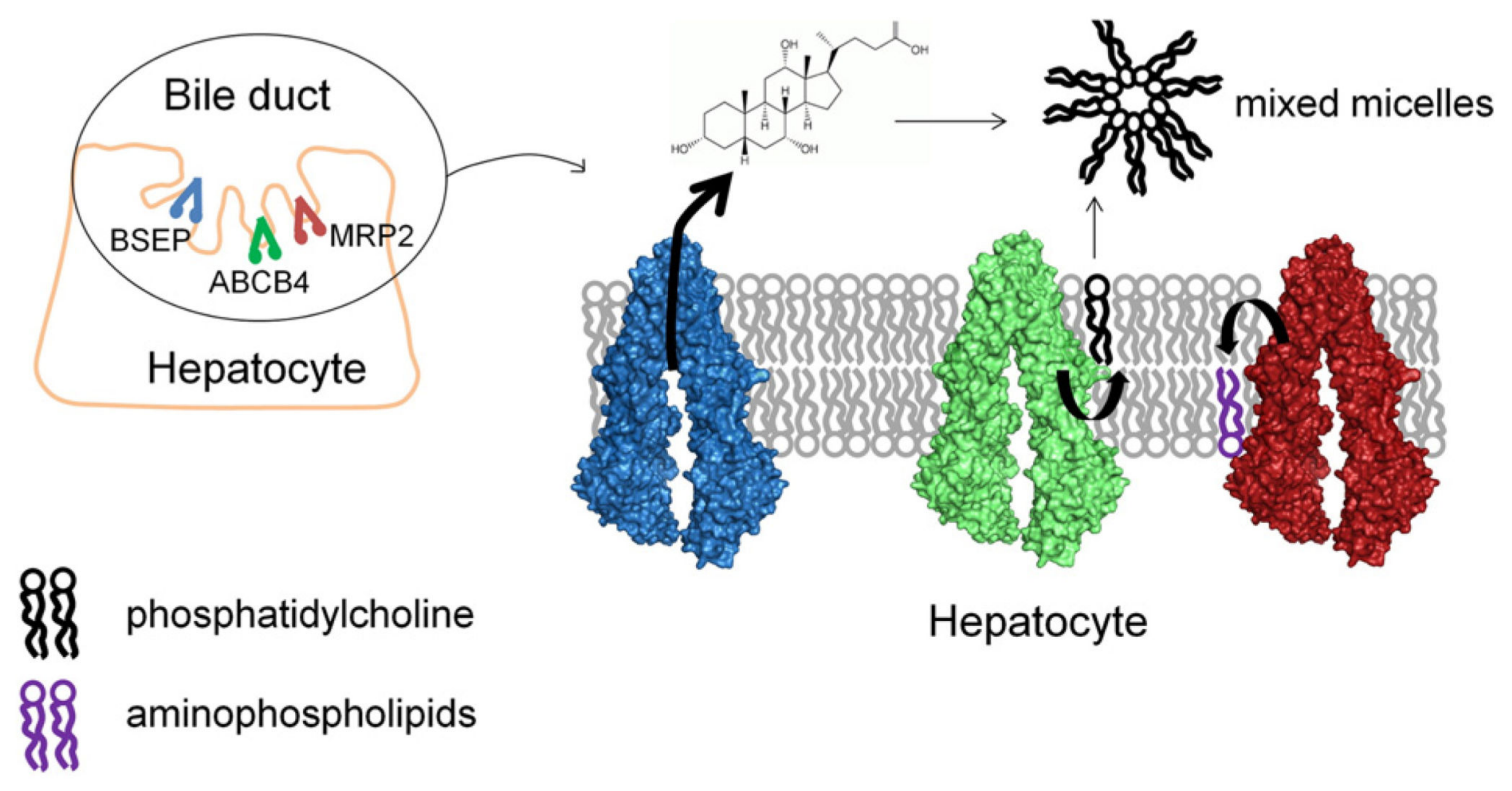
Cooperation of BSEP, ABCB4 and MRP2 in the canalicular membrane of hepatocytes. BSEP (blue) exports the bile salts, ABCB4 (green) flips phosphatidylcholine to the outer leaflet of the membrane, where it is recruited by bile salts to form mixed micelles. MRP2 (red) maintains the asymmetry in lipid composition by flipping aminophospholipids to the inner leaflet of the membrane.

Canalicular ABC-transporters, which mediate the excretion of individual bile constituents, play a key role in bile formation and cholestasis. Some of these constituents, such as bile acids, cause serious damage to hepatocytes and bile duct cells, which might lead to inflammation, fibrosis, cirrhosis, sitosterolemia, hyperbilirubinemia, cholestasis, and potentially also cancer [16,17]. Especially, the proper interplay (see Fig. 1) of the bile salt export pump (BSEP, gene ABCB11) with MDR3 (gene ABCB4) is critical for the formation of bile salt micelles, and inhibition of BSEP has been clearly linked to drug-induced liver injury (DILI) [18]. However, besides BSEP and MDR3, MRP2 (gene ABCC2) as well as P-gp and BCRP are involved. Thus, there are multiple possibilities for drugs and nutrients to interfere with the liver transportome, and we are just beginning to understand how this is linked to hepatotoxicity. One possible starting point are diseases linked to ABC-transporter mutations. For example, homozygous-null MDR3 mutations cause progressive familial intrahepatic cholestasis [19]. MDR3 flops phosphatidylcholine into the bile canaliculus to protect the biliary tree from the detergent activity of bile salts. Thus, a misbalance of BSEP and MDR3 activity leads to toxic concentrations of bile salts either in the hepatocyte or in the bile duct.

### 1.3 Diseases related to malfunction of ABC-transporters

On a more general level, there are numerous diseases which have been linked to improper functioning ABC-transporters. The paradigm example is cystic fibrosis, which is caused by mutations of the CFTR chloride channel [20]. The CFTR chloride channel is encoded by the ABCC7 gene, which is mutated in patients with cystic fibrosis. ATP-driven conformational changes open and close a gate to allow transmembrane flow of chloride anions down their electrochemical gradient. Very recently, Vertex launched a drug which potentiates the function of deltaF508 mutated CFTR and thus compensates for the impaired function. Another compound developed by Vertex acts as pharmacochaperone, thus increasing the concentration of CFTR in the membrane.

Other examples are the link of MRP2 to Dubin–Johnson syndrome [21], of ABCA1 to Tangier disease [22], and of BCRP to gout [23].

Finally, there is increasing evidence that P-gp, BCRP, MRP1 (gene ABCC1) and the cholesterol transporter ABCA1 may contribute to the pathogenesis of Alzheimer's Disease (for a review see [24]). Thus, modulation of their activity might be a new concept for the treatment of Alzheimer.

### 1.4 Drug–drug and drug–nutrient interactions

In an aging society, drug–drug interactions become an extremely important issue. Elderly patients are quite often subject to complex medications, and the risk of severe drug–drug interactions increases with the number of drugs. Most often, these interactions are linked to cytochrome P450-related metabolism of compounds, *i.e.* compound A blocks the metabolism of compound B, which increases the concentration of compound B beyond the toxic level. However, there are numerous reports in the literature pointing towards drug–transporter interaction as additional contributor to severe drug–drug interactions. It could be that compound A blocks a transporter which is transporting compound B, thus influencing the distribution of compound B. Another scenario is that compound A induces the expression of a certain transporter, which then influences the distribution of all substrates of this transporter. A selected example is the interaction of rifampin with the P-gp substrate digoxin, where patients treated with rifampin and digoxin show considerably increased digoxin levels. As the renal clearance and half-life of digoxin was not altered by rifampin, this is most probably due to an increase of the intestinal P-gp content due to an induction of P-gp expression [25].

Another well documented example is the influence of P-gp inhibitors on the distribution of HIV-1 protease inhibitors into brain and testes [26]. However, a very recent study based on a detailed analysis of clinical drug–drug interaction studies revealed that the risk for drug–drug interactions caused by P-gp inhibition is quite limited. A significant risk could only be detected when both P-gp and CYP3A are inhibited [27].

One of the major functions of ABC-transporters is the transport of natural toxins. Therefore, they are definitely also linked to drug–nutrient interactions. One prominent example are flavonoids, which have been shown in numerous studies to interact with P-gp [28–30] and BCRP [31]. Of course, induction of protein expression — like it has been shown for St. Johns wort (Hypericum perforatum) — and cytochromes might also be a major issue, especially when considering that nuclear receptors are involved for both cytochromes and ABC-transporters [32].

Considering the multiple involvement of ABC-transporters in ADMET properties of drugs as well as their potential role as targets for treatment of multidrug resistant tumours, it is evident that numerous computational studies have been performed with the aim to predict potential compound–transporter interaction and to explore the molecular basis of the polyspecificity of these transporters. These started with ligand-based approaches, which extended to structure-based studies when the first X-ray structures became available.

## 2 Ligand-based models

### 2.1 Machine learning approaches for predicting inhibitors of ABC-transporters

**Fig. 2:**
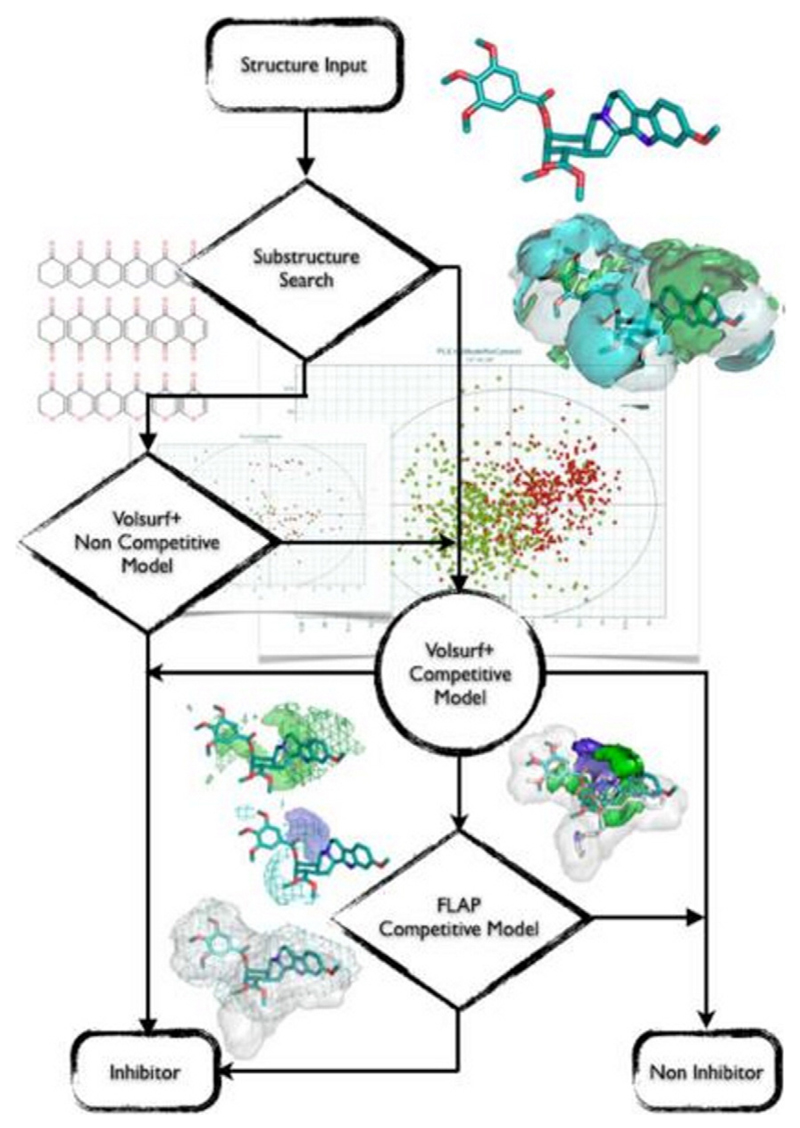
Flow chart of the Composite Model. Reprinted with permission from [40]. Copyright 2011 American Chemical Society.

P-glycoprotein is definitely the paradigm protein for the whole family of ABC-transporters. Thus, basically all methods available for ligand-based design have been applied. These include conventional Hansch analysis, linear and non-linear classification algorithms, pharmacophore modeling, as well as supervised and unsupervised artificial neural networks. There are numerous reviews published which summarize these studies, and the reader is referred to a small selection for further reading [33–38]. However, the challenges in the field of ABC-transporter modeling are manifold, and the main question — what is the molecular basis for the polyspecificity — is still not solved. A large number of chemical scaffolds for inhibitors of P-gp have been published, and, for basically all of them, structure–activity relationships could be derived. This indicates that there are local effects (binding sites?) which translate to a distinct structure–activity relationship (SAR). For each scaffold investigated, clear determinants for high and low inhibitory activity could be established. They most often relate to quite basic physicochemical parameters, such as lipophilicity, H-bonding, aromatic rings, and may be also charge. However, after more than 30 years of intense research, there is still no clear understanding of the molecular basis of compound–transporter interaction which would translate to a set of general rules for medicinal chemists that could help them to enhance or to avoid P-gp inhibitor properties in a lead optimization program. Interestingly, also the concepts of ligand efficiency and lipophilic efficiency have to be applied in a different way than for conventional targets [39]. In recent years, the focus shifted to classification models for large data sets in order to allow *in silico* profiling of compound libraries. Also in this area a number of publications appeared in the literature, and we will just summarize a few recent ones to outline the main strategies followed. One of the groundbreaking contributions is the work of Broccatelli and colleagues [40], who used a combination of molecular field analysis, pharmacophore-based representation of the compounds, as well as physicochemical descriptors to develop both global and local models for P-gp inhibitors. Based on a data set of 1275 compounds derived from 61 references, the authors established a workflow which combines specific (pharmacophore) and nonspecific (general physicochemical) descriptors (Fig. 2). The final model points towards flexibility, hydrophobic surface area, and logP as main discriminating physicochemical parameters for inhibitors/non-inhibitors. Furthermore, shape also emerged as a crucial factor, indicating the importance of the 3D description of the molecules. The authors reported an accuracy of 0.86, specificity of 0.8, sensitivity of 0.9 and Cohen's kappa of 0.7 on a true external set.

Chen and colleagues compiled a large data set from literature, comprising in total 1273 compounds [41]. Their classification approach is based on recursive partitioning and naive Bayes categorization using a set of physicochemical descriptors and various fingerprints. Also in their models, logP is an important contributor to distinguish inhibitors from non-inhibitors. The introduction of fingerprints remarkably improved the prediction accuracy of the models (from a sensitivity of 0.69 and specificity of 0.70 with only physicochemical descriptors on an external test set to a maximum of 0.84 in sensitivity and 0.87 in specificity on the same set but with fingerprints) and furthermore allowed to identify molecular fragments which are favorable or unfavorable for P-gp inhibition. However, one should bear in mind that all methods linking substructures/fragments to biological activity of course heavily depend on the presence/absence of these fragments in the data set. For example, a data set which includes a series of propafenone analogs will of course point towards the importance of an aryloxypropanolamine moiety for P-gp inhibitory potency. However, detailed structure–activity relationship studies showed that the hydroxy-group of the propanolamine seems not to be involved in compound–transporter interaction [42].

While in the case of P-gp datasets of considerable size are available in the literature, for most of the other ABC-transporters there is still a lack of data for establishing *in silico* models. Thus, for MDR3, which is a phospholipid transporter expressed in the liver, only 5 compounds are retrieved by the Open PHACTS Discovery Platform [6], including taxol, vinblastine and verapamil. Considering the fact that MDR3 is the closest homolog to P-glycoprotein (sequence identity 75%), it seems quite unlikely that the protein is inhibited only by five compounds. In case of BCRP, BSEP, MRP1, and MRP2, considerable progress has been made within the past few years, allowing developing *in silico* models.

Very recently, the hitherto largest data set for BCRP has been compiled by Montanari and Ecker, and includes 978 unique compounds extracted from 47 studies [43]. Subsequently, the data set was used to derive a Bayesian classification model using ECFP_6 fingerprints. This allowed extracting important substructures, which are mostly in line with currently published SAR studies around BCRP inhibition. Basically, the number of nitrogen atoms, the aromaticity and the presence of fused aromatic heterocycles seem to favor inhibition, while the presence of sulfur atom, five-membered rings, or amide linkers seems to favor inactivity. The authors report an accuracy of 0.92 and an area under the ROC curve of 0.85 in cross-validation for this naive Bayes model.

In case of the human bile salt export pump (BSEP), Warner *et al*. [44] used a recently described *in vitro* membrane vesicle BSEP inhibition assay to quantify transporter inhibition for a set of 624 compounds. Relating a set of physicochemical properties of the compounds to BSEP inhibition, they showed that lipophilicity and molecular size are significantly correlated with BSEP inhibition. BSEP inhibitor classification by a support vector machine model leads to a total accuracy of 0.87. The model could be further used to minimize the propensity of drug candidates to inhibit BSEP.

In case of MRP2, an ABC-transporter which also might be involved in drug–drug interactions in the liver, Pedersen *et al*. [45] measured a set of 191 structurally diverse drugs and drug-like compounds for inhibition of MRP2-mediated transport of estradiol-17-d-glucuronide (E17G) in inside-out membrane vesicles from Sf9 cells overexpressing human MRP2. Based on these data, a multivariate orthogonal partial least squares discriminant analysis (OPLS-DA) model that distinguishes between MRP2 inhibitors and non-inhibitors was built. The model was capable of correctly classifying 72% of the inhibitors and 71% of the non-inhibitors in the test set. The coefficients in the final model show that a combination of increased lipophilicity, aromaticity, and size is a major determinant for the MRP2 inhibitory effect. Interestingly, the authors also performed an analysis to examine whether inhibitors that have also been reported to be substrates, and which are thus likely to compete with E17G binding at the transport site, were structurally different from other inhibitors. They were indeed on average less lipophilic than other inhibitors and also had a higher molecular weight and a larger polar surface area.

Interestingly, there are also early attempts to perform selectivity profiling studies over several ABC-transporters. For example, Matsson and colleagues [55] used a set of 122 structurally diverse drugs to study the inhibition patterns of P-gp, BCRP, and MRP2. The inhibitor specificities of P-gp, BCRP and MRP2 were shown to be highly overlapping, and a computational model based on multivariate statistics correctly classified 80% of general ABC transporter inhibitors and non-inhibitors in an external test set.

### 2.2 Pharmacophore models for ABC-transporter inhibitors

Of course, in addition to classical QSAR of local compound series and inhibitor/non-inhibitor classification models, numerous pharmacophore models have been derived. This is driven by the aim to understand pharmacophoric and pharmacophobic features which determine ligand–transporter interactions. However, due to the high structural diversity of the ligands, also pharmacophore modeling so far did not lead to a better molecular understanding of the molecular basis of polyspecificity. However, most of the pharmacophore models derived show good capabilities in identifying new ligands with new chemical scaffolds, thus proving their utility. Briefly, Palmeira *et al*. [46] created a pharmacophore model based on 26 known P-gp inhibitors from the flavonoid family, which was then used to screen DrugBank. 167 structures were found to comply with the pharmacophore model with an RMSD of < 1 Å. Out of these 167 structures, 91 fulfilled the Lipinski rules of 5. Finally, 21 compounds were selected for biological testing, whereby 12 were found to significantly increase the intracellular accumulation of Rhodamine-123, a P-gp substrate. Analogously, Pan *et al*. [47] created a pharmacophore model based on 25 BCRP inhibitors and screened the Collaborative Drug Discovery Database, which comprises 2815 FDA-approved drugs selected from all medications on the market since 1938. 33 drugs were tested *in vitro* for their inhibitory effects on BCRP-mediated transport of [3H]-mitoxantrone in MCF-7/AdrVp cells, and 19 compounds were identified with significant inhibitory effect on BCRP transport function. For BSEP, a small set of 5 compounds served as basis for a pharmacophore model, which was validated against a set of 59 compounds, including registered drugs. The model recognized 9 out of 12 inhibitors, which could not be identified based on general parameters (such as molecular weight or SlogP) alone. Finally, the model was used to screen a virtual compound database of commercially available compounds. A number of compounds found via virtual screening were tested and displayed statistically significant BSEP inhibition, ranging from 13 ± 1% to 67 ± 7% of control (P < 0.05) [48].

A pharmacophore for MRP1 was recently built on five diverse and potent inhibitors [36]. It is composed of 3 aromatic rings and 3 H-bond donor features and was able to retrieve 3 known inhibitors of MRP1 among a large database of 500 drugs. For MRP2, Zhang and colleagues [49] reported a pharmacophore built on nine potent and diverse inhibitors. It contains two H-bond acceptor features and one hydrophobic feature. The model gave a sensitivity of 78% and a specificity of 70%, with an overall accuracy of 74%.

### 2.3 Models for predicting substrates

While for inhibitors a set of assays is available which leads to precise IC_50_ values, the case of substrates is much more complicated. Most commonly a polarized transport assay across a monolayer of cells overexpressing a distinct transporter is used. Thus, the *in silico* models derived on basis of these data mostly use binary classification algorithms (substrate versus non substrate). To our knowledge the largest data set for P-gp substrates/non-substrates in the public domain was compiled by Li *et al*. [50] (423 substrates, 300 non-substrates). Analyzing the distributions of eight basic physicochemical properties for the substrates and non-substrates showed that molecular weight and solubility are the main factors differentiating P-gp substrates from non-substrates. When comparing the 423 substrates with a set of 735 P-gp inhibitors, inhibitors proved to be significantly more hydrophobic than substrates while substrates tend to have more H-bond donors than inhibitors. Applying a naive Bayes classifier using a set of simple molecular properties, topological descriptors, and molecular fingerprints, a classification model with very good performance was retrieved (Matthews correlation coefficient (MCC) = 0.824, prediction accuracy = 91.2% for leave 20% out cross-validation, prediction accuracy of 83.5% for a test set of 200 molecules). The most important structural fragments provided by the Bayesian classifier indicate that H-bond acceptors arranged in distinct spatial patterns as well as flexibility are quite essential for P-gp substrate-likeness.

In another setting, Wang *et al*. [51] used a set of 332 compounds to develop a classification model using support vector machine. The best model (MCC = 0.73) shows a prediction accuracy of 0.88 on a test set. Examination of the model based on ECFP_4 fingerprints revealed substructures such as nitrile and sulfoxide, which have a higher frequency in non-substrates than in substrates. However, as already previously stated, this should be taken with caution, as substructure analysis depends on the occurrence of the respective fragments in the training set. Also for BCRP, a larger set of 263 substrates and non-substrates has been collated from literature and classified via a support vector machine [52]. The final SVM model had an overall prediction accuracy of 73% for an independent test set of 40 compounds and was integrated to a free web server (http://bcrp.althotas.com).

While finding a large dataset for MRP2 substrates and non-substrates is not an easy task to date, Pinto and colleagues [53] made use of a fuzzy dataset correlating transporter expression in cancer cell lines with the substrate capability of the tested compounds [54] to build classification models predicting MRP2 substrates. The authors reached a sensitivity of 0.77 and specificity of 0.72 in their best settings where 16 physicochemical descriptors were used to build a cost sensitive Random Forest. This technique allowed taking into account the imbalance of the data by penalizing predictions errors made by the model on the minority class.

In summary, there are numerous models published which are able to predict inhibitors and/or substrates of the most important ABC-transporters. All the models described here are summarized in Table 1. However, a set of general rules with respect to the main driving factors for ligand–transporter interaction, which go beyond lipophilicity, size, and H-bonding, is still missing. Furthermore, most of the models lack proper applicability domain assessment, which renders it difficult to judge their performance in a broader chemical space. Finally, when checking the original publications where the data were coming from, it becomes evident that numerous different assays are used to measure ABC-transporter inhibition. Thus, an upfront careful curation of the data seems mandatory before using them for large scale models.

## 3 Data curation

Working with large datasets seems to be the way to build high quality models and derive general trends for compounds interacting with ABC-transporters. Data, however, is scarce, at least for some less studied transporters. Typical medicinal chemistry studies report bioactivities for a small set of chemically related compounds. Scientists wanting to build large datasets must collect and merge together such data, using databases like ChEMBL [56] and Pubchem [57], but also manual search through MEDLINE. Now, what if the groups measuring ABC-transporters substrate or inhibition activities each use their own assay design? Then merging together data becomes a challenging task. Zdrazil *et al*. [58], studied all bioassays from ChEMBL for P-gp inhibition and transport, when these assays reported IC_50_, EC_50_ or Ki values. Subsequently, they annotated assays according to their potential for being combined together in a large QSAR dataset. The results show the importance of overlapping binding sites for the different substrates used in the bioassays, as well as the cell line in which the transporter is expressed.

In another recent study [59], the authors compared IC_50_ values obtained across several laboratories for P-gp inhibition, each using several assay methodologies. The variability range was over 20 fold for all compounds tested, and the study concluded that the most important actor was inter-laboratory variability rather than inter-assay variability. Beyond the assay diversity and inter-laboratory variabilities, Balimane and colleagues [60] have pointed out yet another source of variability, namely the calculation of inhibition given one raw set of data measured on one assay by one laboratory. It seems that, depending on the calculation method used, one can draw entirely different conclusions regarding the inhibition capability of compounds.

For other less studied transporters like BCRP, the picture gets worse: most of the assays use different cell lines, and little is known about the binding sites of the different substrates used in these assays [61]. The resulting problem is that a compound may show activity in an assay with a given substrate, but no activity in the presence of another substrate. One solution is to compare activities reported across distinct assays, exclusively use the data for building classification models, apply a threshold for activity assay by assay and remove problematic compounds [43].

To alleviate the aforementioned problems related with bioactivity data in the field of transporters, one could propose some simple measures to apply and change the current habits in the field. While agreeing on a specific assay methodology seems a bit irrealistic, a set of reference compounds for each transporter could be defined (both inactives and actives) and recommend that each laboratory willing to publish new bioactivity data must also report the bioactivities obtained on their assay for this group of reference compounds. That way, inter-laboratory and inter-assay differences would be immediately spotted and taken care of appropriately when merging data from different sources.

## 4 Structure-based models

Due to the tremendous progress in the field of structural biology, structures of transmembrane transporters, including several ABC-transporters, became available. Most of them were from prokaryotes, and only very recently structures from eukaryotic organisms were also resolved in a resolution which allows starting structure-based approaches. However, the only human ABC-transporter crystallized so far is ABCB10 [62]. Nevertheless, the whole field of ABC-transporter research was inspired by the first structures being deposited in the Protein Data Bank [63], and protein homology models of P-gp immediately became available.

More recent templates available for homology modeling are provided in Table 2.

**Fig. 3:**
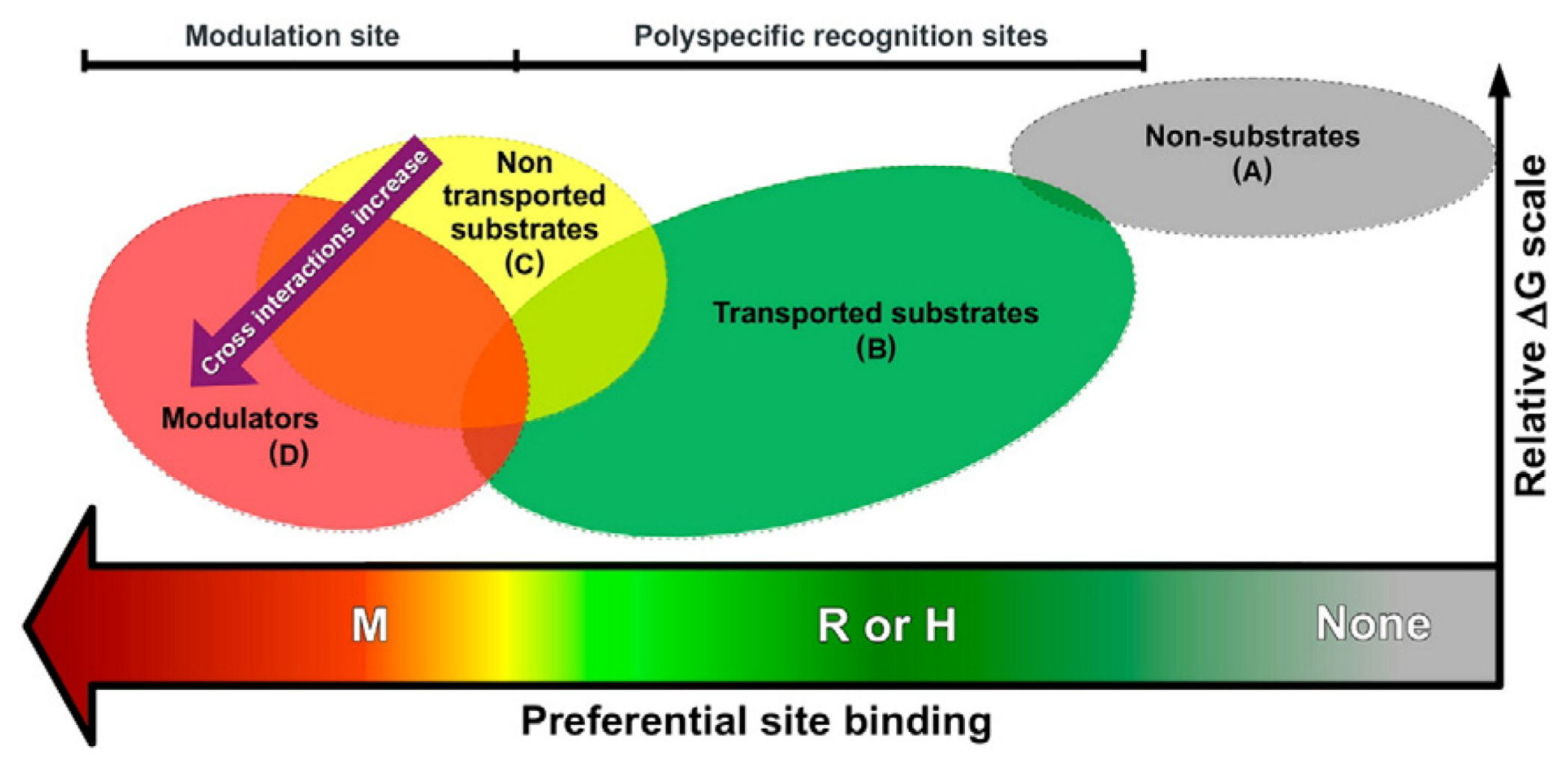
Classification scheme for P-gp substrates. Reprinted with permission from [78]. Copyright 2013 American Chemical Society.

Although most of these structures are in sufficient resolution to serve as templates for structure-based studies, one needs to bear in mind that there is still no protein structure cocrystallized with a classical substrate/inhibitor, such as verapamil or cyclosporin. The only structure which includes a small molecule is the one from mouse P-gp [65]. Furthermore, the transporters undergo a substantial conformational change when progressing through the transport cycle, which renders all structures available only snapshots of a very distinct point in the whole conformational space. Nevertheless, especially for P-gp, numerous docking studies of selected ligands into homology models were performed with the aim to understand the molecular determinants of binding (for reviews, see *e.g.* [33,34,35,72,37]). However, experimental validation, especially with respect to prospective validation of the binding hypotheses retrieved, is mostly missing. In this section we will thus focus on recent advances where structure-based studies were used more in the sense of virtual screening rather than providing distinct poses for selected compounds. Dolghih *et al*. [73] implemented a flexible receptor docking protocol for docking a set of 26 drugs known to interact with P-gp. 102 endogenous metabolites, assuming that they will not interact with the transporter, served as negative control. As a template, mouse P-gp bound to the cyclic peptide QZ59-RRR was used. Subsequently, the dataset from Doan *et al*. [74] of FDA-approved drugs that included results of the monolayer efflux and CAM inhibition assays, was used for docking. The results suggest that many P-gp substrates bind deeper in the cavity than the cyclic peptide in the crystal structure, and that specificity in P-gp is better understood in terms of physicochemical properties of the ligands (and the binding site), rather than being defined by specific sub-sites. Klepsch *et al*. [75] also built on the mouse P-gp complexed with QZ59-RRR and implemented a docking protocol which exhaustively samples the pose space of a small set of ligands which show a distinct SAR pattern followed by common scaffold clustering. The SAR information is then used to priorities the pose cluster in order to retrieve an experimental-data-guided binding hypothesis. Subsequently, the docking protocol was used to classify a large set of 1608 inhibitors and non-inhibitors of P-gp. Although the performance of the structure-based classification was considerably lower (61% for the external test set) than those obtained by Random Forest or SVM classification (73% and 75%, respectively), it shows that structure-based classification of ligands of ABC-transporter is within reach [76]. A very comprehensive approach was used by Ferreira *et al*. [77]. Using a previously refined structure of murine P-gp, they characterized the M-, H- and R-site by means of molecular docking. The drug-binding pockets were defined as substrate- or modulator-binding sites according to the molecules that preferentially docked in each location. For the authors, “modulator” refers to compounds that appear to block the efflux of substrates. The substrate-binding sites H and R refer to Hoechst 33342 and rhodamine-123, respectively. Analogously, the modulator-binding (M) site was linked to the main interaction site for verapamil. Subsequently, they carried out further docking studies with molecules classified as substrates or modulators in order to retrieve a structure-based classification model with the ability to discriminate substrates from modulators. The classification scheme contains four main categories: (i) non-substrates, (ii) transported substrates, (iii) non-transported substrates, and (iv) modulators (Fig. 3). Their model properly predicted 14 modulators out of 19 (74%), 20 substrates out of 32 (63%) and 2 out of 3 non-substrates. However, the authors rightly conclude that “…. *the substrate-binding sites may present different characteristics at different steps of the ef*fl*ux mechanism, possibly interconverting the H-site and the R-site in one another, partially explaining the induced-*fi*t and polyspeci*fi*city models proposed for Pgp substrate recognition*”, and point towards the importance of molecular dynamics simulations for further insights into the dynamics of the protein.

In a different publication [78], the authors indeed performed 100 ns molecular dynamics simulations in order to refine their homology model. Subsequent 20 ns production runs with a small set of ligands indicated that the number of interactions established between several ligands and the drug binding pocket might allow distinguishing inhibitors from substrates. Indeed, the modulators studied consistently established a higher number of nonbonded interactions, mainly aromatic ones, when compared with substrates. In the particular case of verapamil, the increased nonbonded interactions established, which is also shown by the modulator tariquidar, classifies the molecule as a modulator. This is well in accordance with a previously developed pharmacophore [79], where the ability to establish a greater number of hydrophobic interactions within the pocket is one of the major features that allows a molecule to block competitively the substrate binding. These studies convincingly demonstrate that structure-based modeling in the field of ABC-transporter has become a valuable tool for a deeper understanding of the molecular features driving ligand–transporter interaction. However, almost all studies focus on P-gp. This is mainly due to the fact that both mammalian structures available are from transporters belonging to the B-family (P-gp and ABCB10). The C-family has the so-called TMD 0, which consists of 5 transmembrane helices (thus having 5 + 12 transmembrane helices) where no suitable template exists. Even worse is the case of members of the G family (BCRP), which show a reversed order of nucleotide binding domain and transmembrane domain. Although there is a considerable substrate and inhibitor overlap between P-gp and BCRP, there is no suitable template for modeling the whole transporter. Thus, new structures are heavily awaited by the community. These might well use also other methods than X-ray crystallography, such as the one of TmrAB published by the Tampe group in November 2014 [80].

## 5 Future challenges

Challenges in the field of ABC-transporter are manifold. With respect to the prediction of drug transporter interaction, there are on our point of view several immediate issues which should be mentioned. The most obvious one is the availability of an atomic resolution structure of human P-glycoprotein in complex with a prototype ligand such as verapamil. This would allow benchmarking all docking studies on this structure, which definitely would increase the validity of the binding hypotheses retrieved. Nevertheless, in early drug discovery, *in silico* models based on machine learning will still be the main tools for prioritization of large compound libraries. *In silico* classification models will only show high predictivity if the underlying data are of high quality and of a considerable size. In case of P-glycoprotein, the size of the datasets available in the public domain is sufficient, but the use of almost 50 different assays currently does not allow combining all the data and to compile a large, high quality dataset for training the models. For ABC-transporters other than P-glycoprotein, the situation is even worse, as already the available datasets are small. For solving the issue of different assays, a transporter assay ontology combined with the definition of a set of standard reference compounds would be highly recommended.

Another important issue is the flexibility inherent to P-gp and most probably to all transmembrane transporters [81]. Even if there would be a set of crystal structures, they still would only cover a small portion of the conformational space of the transporter. With the ever increasing computing power, also pushed by GPU clusters, large grids and special computer hardware, molecular dynamics simulations of transmembrane proteins in the ms range are possible. Simulating an ABC-transporter through the whole transport cycle and validating/constraining the simulation by respective biophysical and biochemical experiments seems already feasible. Once the system is established, this would also allow including small molecules, be it substrates, inhibitors, or modulators. However, also the composition of the membrane, its cholesterol content, as well as the behavior of the ligand in the membrane needs to be considered [82]. This opens another layer of complexity for full atomic simulations. Another issue linked to dynamics is the on- and off-kinetics of the ligands. There is increasing evidence that the dissociation kinetics of a given drug from its target (its residence time) may be more relevant for the *in vivo* efficacy than its *in vitro* equilibrium binding constant. Recent examples demonstrate that receptor subtype selectivity might be driven by differences in dissociation kinetics rather than by affinity differences [83]. We have first evidence in our lab that this is also the case for propafenone-type inhibitors of P-gp, and also other groups already speculated on this [84].

The importance of ligand–transporter interaction for prediction of toxicity and safety needs much more attention. As outlined in the introduction, ABC-transporters play a major role in toxicity related to drug–drug interaction. However, due to the multiple interplay of several transporters, this requires more complex strategies. In might well be that there are a sort of redundant backup systems, where one transporter can compensate for the functional failure of another one. A well established example is the interplay of P-gp and BCRP at the blood–brain barrier. But also in the liver there are numerous ABC-transporters, which have to be considered. There are already a few publications available which attempt to simultaneously predict the interaction of a compound of interest with several transporters (not necessarily only ABC-transporter; see *e.g.* [85]). Selectivity profiling, of course, is strongly linked to the availability of proper data, as one would need a matrix of a set of compounds measured at all transporters of interest. These models could then be further used for linking *in vitro* interaction profiles to *in vivo* effects, as recently has been shown for a set of antidepressant drugs and their side effects observed in clinical studies [86]. This would allow including ligand–transporter interaction profiles into very early safety considerations and help to reduce late stage failures in clinical studies.

## 6 Conclusions

ABC-transporters represent an integral and important part of the human transportome. Although there are only 49 genes described in humans, they fulfill important roles and are strongly linked to drug absorption, distribution, and elimination. Furthermore, besides cytochromes, they are also involved in drug–drug interactions and thus also toxicity of drugs. With the increasing accessibility of biological data and the tremendous progress of structural biology, our understanding of the molecular basis of ligand–transporter interaction is progressing. In this review, we have outlined recent ligand-based models built on large datasets rather than on congeneric series. While these models allow screening rapidly large databases of molecules to predict their substrate or inhibition properties, their interpretation remains at the level of substructures or general physico-chemical properties. On the structure-based side, the presence of crystal structures of the B subfamily allowed building high quality homology models for P-gp and a mapping of different binding sites has started. Such advances have not yet been noted for other ABC-transporters, but we believe that new structures will appear in the PDB that will allow similar studies to be performed.

However, the influence of on- and off-kinetics of ligands on their efficacy as well as the multiple interplay of ABC-transporters under *in vivo* conditions pose additional challenges which the community will face in the near future.

## Figures and Tables

**Table 1:** Summary of all ligand-based models described in Section 2.

Transporter	Type of model	Dataset^a^	Predictivity	Publication
P-gp	Combined	1275 inhibitors	Accuracy: 0.86	Broccatelli *et al*. [40]
P-gp	Naive Bayes	1273 inhibitors	Sensitivity: 0.835 Specificity: 0.866	Chen *et al*. [41]
P-gp	Pharmacophore	26 inhibitors	12/21 tested were active	Palmeira *et al*. [46]
P-gp	Naive Bayes	723 substrates	Accuracy: 0.84	Li *et al*. [50]
P-gp	SVM^b^	332 substrates	Accuracy: 0.88	Wang *et al*. [51]
BCRP	Naive Bayes	978 inhibitors	Accuracy: 0.92	Montanari and Ecker [43]
BCRP	Pharmacophore	25 inhibitors	19/33 tested were active	Pan *et al*. [47]
BCRP	SVM^b^	263 substrates	Accuracy: 0.73	Hazai *et al*. [52]
BSEP	SVM^b^	624 inhibitors	Accuracy: 0.87	Warner *et al*. [44]
BSEP	Pharmacophore	5 inhibitors	Sensitivity: 0.75	Ritschel *et al*. [48]
MRP2	OPLS-DA^c^	191 inhibitors	Sensitivity: 0.72 Specificity: 0.71	Pedersen *et al*. [45]
MRP2	Pharmacophore	9 inhibitors	Accuracy: 0.74	Zhang *et al*. [49]
MRP2	Random Forest	1204 substrates	Sensitivity: 0.77 Specificity: 0.72	Pinto *et al*. [53]
MRP1	Pharmacophore	5 inhibitors	Not clear	Chang *et al*. [36]
P-gp, BCRP, MRP2	PLS-DA^d^	122 inhibitors	Accuracy: 0.8	Matsson *et al*. [55]

a Size and type of data (for models that are not pharmacophores, both active and inactive are present).

b Support vector machine.

c Orthogonal partial least squares discriminant analysis.

d Partial least squares discriminant analysis.

**Table 2:** Existing 3D structures of ABC transporters in the Protein Data Bank (PDB).

Year	Publication	PDB IDs	Species	Protein	Res.^a^	State
2012	Jin *et al*. [64]	4F4C	*C. elegans*	Pgp-1 (Uniprot: P34712)	3.4 Å	Open-in
2012	Shintre *et al*. [62]	3ZDQ, 4AYT, 4AYX, 4AYW	*H. sapiens*	ABC transporter 10 protein (Uniprot: Q9NRK6)	2.85 Å	Open-in
2009	Aller *et al*. [65]	3G5U 3G60 3G61	*M. musculus*	MDR1A (Uniprot: P21447)	3.8 Å	Open-in
2013	Ward *et al*. [66]	4KSB 4KSC 4KSD 4LSG	*M. musculus*	MDR1A (Uniprot: P21447)	3.8 Å	Open-in
2014	Li *et al*. [67]	4M1M, 4M2S, 4M2T	*M. musculus*	MDR1A (Uniprot: P21447)	3.8 Å	Open-in
2007	Dawson and Locher [68]	2ONJ	*S. aureus*	SAV1866 (Uniprot: Q99T13)	3.4 Å	Open-out
2006	Dawson and Locher [69]	2HYD	*S. aureus*	SAV1866 (Uniprot: Q99T13)	3.0 Å	Open-out
2007	Ward *et al*. [70]	3B5Y, 3B5Z, 3B60	*S. typhimurium*	Permease protein msbA (Uniprot: P63359)	3.7 Å	Open-out
2012	Hohl *et al*. [71]	3QF4	*T. maritima*	Uncharacterized ABC transporter (Uniprot: Q9WYC4)	2.9 Å	Open-in

a Resolution. When more than one PDB ID is given, the lowest resolution is reported.
